# Short-term effects of the COVID-19 state of emergency on contraceptive access and utilization in Mozambique

**DOI:** 10.1371/journal.pone.0249195

**Published:** 2021-03-25

**Authors:** Jessica Leight, Catherine Hensly, Marcos Chissano, Liza Ali

**Affiliations:** 1 International Food Policy Research Institute, Washington, DC, United States of America; 2 Department of Economics, American University, Washington, DC, United States of America; 3 Population Services International, Maputo, Mozambique; Johns Hopkins Bloomberg School of Public Health, UNITED STATES

## Abstract

The COVID-19 pandemic has increasingly disrupted the global delivery of preventive health care services, as a large number of governments have issued state of emergency orders halting service delivery. However, there is limited evidence on the realized effects of the pandemic and associated emergency orders on access to services in low-income country contexts to date. To address this gap, this paper analyzes administrative data on utilization of contraceptive health services by women referred via community health promoters in two large urban and peri-urban areas of Mozambique. We focus on the period immediately surrounding the national state of emergency declaration linked to the COVID-19 pandemic on March 31, 2020. Data reported for 109,129 women served by 132 unique promoters and 192 unique public health facilities is analyzed using logistic regression, interrupted time series analysis and hazard analysis. The results demonstrate that the imposition of the state of emergency is associated with a modest short-term drop in both service provision and utilization, followed by a relatively rapid rebound. We conclude that in this context, the accessibility of reproductive health services was not dramatically reduced during the first phase of the pandemic-related emergency.

## Introduction

The onset of the COVID-19 pandemic in March 2020 has disrupted the delivery of both primary and preventive health care services across the globe. This disruption reflects a range of complex factors, but one particularly important cause is the imposition of government restrictions (states of emergency, travel limitations, and other related measures) that limit the provision of health services. In the developing world especially, these pandemic-related shocks may stall recent gains in enhancing access to primary health care, especially for vulnerable women and children [1, 2]. Progress toward the related Sustainable Development Goals (namely goal 3.1, reduction of the global maternal mortality ratio, and goal 3.2, reduction of neonatal and under-5 mortality) may be at risk [3].

Previous evidence shows similar disruptions in health services during the Ebola outbreak in 2013–2016 generated an additional burden of maternal and child deaths comparable to the direct mortality costs of the Ebola outbreak itself [4]. Though clearly the current pandemic is very different, early estimates suggest that indirect effects related to COVID-19 could potentially result in hundreds of thousands of excess maternal and child deaths [5].

More specifically, in the area of reproductive health care, the COVID-19 shock is projected to cause a substantial diversion of resources [1], leading policymakers to call for a focus on the importance of reproductive health [6]. The imposition of lockdowns and emergency measures may have diverse effects on the provision of contraceptives and associated services. On the supply side, facilities may close, supply chains for health commodities may be disrupted, or health workers may experience higher levels of absenteeism, reducing the availability and accessibility of reproductive services and contraception in particular [7]. On the demand side, women may perceive the risk of infection from visiting a health facility to be too high to justify accessing contraception, or may face reduced time availability or a shortage of economic resources that render it challenging for them to utilize care [4]. In this case, women may avoid health facilities entirely.

Much of the literature around the effects of COVID-19-linked restrictions remains speculative, however, since many methods of data collection from service users have been suspended due to the risk of disease exposure and transmission. Nearly all national statistic offices surveyed by the UN in May 2020 had at least partially halted face-to-face data collection, and every low-income country respondent reported at least moderate effects from the pandemic on their ability to meet international reporting requirements [8]. As a result, there is limited evidence around the realized effects of the COVID-19 pandemic and related governmental response on access to reproductive health care, particularly in low-income country contexts such as Mozambique.

On March 31, 2020, the Mozambican government declared a state of emergency in response to the COVID-19 pandemic. On that day, Mozambique had only eight confirmed cases of COVID-19 but was characterized by the WHO as experiencing local transmission of the virus [9]. Provisions of the state of emergency included the closure of all schools; the closure of entertainment establishments and suspension of entertainment and sports events; the suspension of religious services; a reduction at other workplaces to one third staff capacity; a reduction of public and private transportation to one third the passenger capacity; and limited hours at markets [10]. In addition, a 14-day quarantine was imposed on all individuals entering the country, and the land border with South Africa was almost completely closed [11]. The state of emergency had the potential to broadly affect the provision of health services and health-related programs. This paper focuses in particular on its effects on the Integrated Family Planning Program (IFPP), a reproductive health program funded by USAID.

The IFPP program is designed to improve the accessibility of modern contraceptives in three provinces of Mozambique, with the goal of increasing access to contraception for up to 565,000 new users between 2016 and 2021 [12–14]. This analysis is conducted in partnership with Population Services International (PSI), an organization providing IFPP services within the urban and peri-urban areas of two provinces, Nampula (including Nampula city, Angoche, Ilha de Mocambique, Murrupula and Nacala Porto) and Sofala (including Beira and Dondo) [12, 13]. IFPP services entail the deployment of local community health workers ("promoters”) who offer reproductive health counseling about family planning to women in one-on-one settings in homes and communal areas [12, 13]. Accordingly, the role of promoters can be summarized as information provision and demand creation [22].

The objective of this paper is to analyze the evolution of contraceptive services utilization as accessed through this door-to-door community health worker program in the period immediately surrounding the state of emergency declaration linked to the COVID-19 pandemic in Mozambique. The analysis utilizes real-time administrative data to investigate the effects of the state of emergency on both promoter activity and women’s utilization of facility-based services.

## Materials and methods

### Study context

Mozambique is characterized by a relatively high fertility rate and low contraceptive prevalence rate, similar to other sub-Saharan African countries. The national total fertility rate was 4.9 children per woman in 2018 compared to an estimated rate of 4.7 children per woman for sub-Saharan Africa overall that year [15]. The most recent available estimates from the Mozambique National Malaria and HIV Indicator Survey 2015 suggest that the national contraceptive prevalence rate was 27 percent that year [16], compared to 29 percent in sub-Saharan Africa overall in 2019 [17]. Meanwhile, national unmet need for family planning in Mozambique was estimated as 23 percent in 2015 [16], slightly higher than the median of 17 percent for sub-Saharan Africa overall in 2019 [18].

This analysis focuses on women beneficiaries of the IFPP program, resident in urban and peri-urban areas of Sofala and Nampula provinces. Levels of education and socioeconomic status in this context are generally low: in Sofala, 26% of urban adults report no education and 43% report primary education, while in Nampula the corresponding figures are 36% and 41% [19].

The IFPP program is centered around services delivered by community-based promoters. Promoters provide counseling around family planning to women in their homes or other community locations. Following the initial session, promoters may meet with beneficiaries up to three additional times over the subsequent months to provide follow-up counseling, depending on the individual woman’s needs (i.e. if she is a new user with a previously unmet need, or a continuing user changing methods based on the information received during the first session) [20, 21]. Promoters set their own schedules, but evidence from the data that will be analyzed in this paper suggests the average promoter conducts 13 to 15 sessions per day. Promoters are compensated per session (regardless of outcome) and per referral resulting in a facility visit.

In any given month, there are typically 100–120 active promoters in the target areas of Nampula and Sofala, and all promoters are women of reproductive age residing in the same community in which they work. Promoters do not supply contraceptive methods directly; rather, they conduct adaptive counseling sessions focused on building relationships and providing credible information on available family planning methods and debunking common myths (i.e. “women who use the pill and injection for many years have difficulty becoming pregnant” or “family planning means not having any children”). At the conclusion of each session, promoters provide women expressing demand for family planning with referrals to public health facilities where services are available at no cost. (A subset of these facilities also receive additional IFPP support for services offered.)

### Data

The data employed is collected by PSI, but only de-identified data excluding any identifying information linked to participants were accessed by the research team. Ethical approval was provided by the Comité Nacional de Bioética para Saúde in Mozambique for the broader evaluation of which this data analysis is a part, and written informed consent was obtained from respondents enrolled in the evaluation. (For minors, consent was not obtained from parents given the objective of maintaining confidential their possible interest in contraception, a protocol approved by the ethics board.)

All data are collected via a comprehensive mobile application utilized by promoters and nurses to track activities and referrals. Promoters record the details for each counseling session conducted in the application, including whether a referral was issued. Promoters also capture some basic demographic data about each woman: a categorical variable for age (15–20, 20–25, etc.), whether the woman is a current contraceptive user (and if so, of what method), and whether she reports access to a mobile phone. No other information about demographic characteristics, obstetric or medical history, or knowledge, attitudes, or preferences around contraceptive use or fertility is collected. Data is uploaded to the cloud storage system in real time following the conclusion of the counseling session, and each record is then stamped with the date, time, and GIS coordinates [22, 23].

If the referred woman visits a health facility, this visit is similarly digitally recorded and linked to her referral and the date of her promoter session. These facility records include information about whether the woman received a contraceptive method and are time-stamped. However, program audits and field observations by program supervisors suggest that the timing of when these visits are recorded varies slightly by facility. While many facilities record visits daily and in near real-time, some collect the information and wait to batch-enter records for multiple days at a time, resulting in heaping of the timestamp entries.

In total, the analysis includes 109,129 women served by 132 unique promoters and 192 unique public health facilities in Nampula and Sofala over the time period of January 21 to May 20, 2020. Structural programmatic changes (e.g. changes to promoter incentives, number of required sessions per beneficiary, etc.) complicate attempts at comparisons with preceding months and years; this analysis therefore focuses on the months immediately before and after the state of emergency declaration on March 31, during which time no other major programmatic changes occurred. In addition, the period in early January was characterized by substantial disruptions linked to the return to work activities post-holidays and the presidential inauguration in Mozambique (held January 15). Accordingly, we begin our analysis on the Tuesday following the inauguration, January 21, to minimize this noise.

### Analysis

The analysis focuses on three variables: daily sessions conducted per promoter, the referral rate for promoters, and the contraceptive receipt rate. The first two variables capture promoters’ behavior. They include the daily count of sessions conducted per promoter and the referral rate (the percentage of sessions in which the promoter issues a referral to a facility). These variables enable us to identify whether promoters decreased their activities following the state of emergency declaration, a shift that could reflect promoters’ fear of interacting with members of the public. In addition, promoters may alter their decisions about whether or not to provide referrals to women if they believe that visiting facilities is a risky activity that they should not recommend post-emergency (for example, due to the risks of disease exposure, disrupted services at the clinic, etc.)

The third variable captures the behavior of women participating in the program; the contraceptive receipt rate is defined as the percentage of referred women who access a prescribed method of contraception at the facility. One important finding is that throughout this period, a facility visit is virtually synonymous with receipt of contraception, as 96% of women who visit a facility receive a contraceptive method. For some analyses, we truncate the sample to focus on women who interacted with a promoter on or before May 6, two weeks prior to the final date observed in the sample, and restrict the variable definition to focus on contraceptive receipt within 14 days. The selection of the 14-day follow-up period is informed by data: in the period prior to the state of emergency, the vast majority of contraceptive receipt following a referral to a clinic is observed to be rapid. Based on women visited by promoters prior to April 1, slightly over half of women who visit a clinic following a referral do so within three days, and 75% do so within two weeks.

The objective of analyzing variables linked to both promoters’ behavior (daily sessions per promoter, contraceptive referral) and women’s behavior (contraceptive receipt) is to provide an overview of the effects of the declared state of emergency on both the supply and demand of contraceptive services. Promoters’ behavioral changes are informative about shifts in the supply of services. Behavioral changes observed among women who are visited by promoters are informative about shifts in the demand for services.

We provide graphical evidence of trends, simple regression evidence, an interrupted time series analysis and a hazard analysis to analyze whether there has been a decline in service intensity post-emergency. To facilitate interpretation and reduce volatility, graphical trends have been smoothed using a uniformly weighted moving average unless otherwise noted; in addition, the graphs do not include public holidays and weekends, since promoter activity is minimal during non-working days. Smoothing is applied using a seven-day moving window, including the date itself plus three days prior and three days following the date. (The use of the uniformly weighted moving average also addresses the heaping in recorded dates of facility visits.) The vertical lines on the graphs highlight the first day (April 1) and subsequent extension (April 29) of the national emergency.

The regression analysis is conducted using data at the level of the woman. The dependent variables include a binary variable equal to one for contraceptive referral (Ref_it_), and a binary variable equal to one for contraceptive receipt (Cont_it_) for woman i visited by a promoter on day t. These variables are regressed on a binary variable for post-April 1 (post the state of emergency), Post_t,_, without adjusting for other individual characteristics. In the second specification, the post variable is interacted with the three individual characteristics reported in the data: a binary variable for whether the user reports access to a phone, a binary variable for whether she currently uses contraception, and a binary variable for age under 25. These characteristics are denoted X_it_ for concision.

These three variables are hypothesized to moderate women’s response to the state of emergency. (Given limited evidence in the existing literature that would allow us to conclude how these characteristics may moderate the relationship between the SOE and contraceptive utilization, this analysis should be considered to be exploratory.) Reported access to a phone is a proxy for socioeconomic status as well as access to information. The negative association between the state of emergency and contraceptive receipt is hypothesized to differ based on socioeconomic status; women characterized by higher socioeconomic status and more access to information are hypothesized to show a more limited or null response to the state of emergency. Similarly, we expect a differential response to the state of emergency for women who are current users (who have a demonstrated demand for contraception), and women over the age of 25 (who are more likely to have a larger number of living children, and thus have a higher presumed demand for contraception). In both cases, the hypothesis is that women with these characteristics will not respond to the state of emergency.

All specifications are estimated using logistic regressions clustering standard errors at the level of the week; estimation is conducted in Stata version 14. All regressions are estimated both using the full set of data available (1/21-5/20) and for a restricted time period encompassing the six weeks immediately surrounding the state of emergency (3/1-4/15). The latter restricted sample allows for the identification of the short-term effects of the declaration of the state of emergency.

The interrupted time series analysis is conducted using the same data and examining the variables contraceptive referral and contraceptive receipt. These variables are regressed on a continuous time variable (capturing days elapsed since the first day observed in the data, January 21); a binary variable for Post-April 1; and the interaction of Post-April 1 and the time trend. The binary variable captures the shift in the intercept post-April 1, and the interaction captures the shift in the trend in the post period. The regression is estimated employing ordinary least squares and including as additional covariates the three demographic variables described above (binary variables for young, current user, and reported phone access), and fixed effects for province, promoter and neighborhood. Standard errors are clustered at the level of the week.

Finally, we estimate a Cox proportional hazards model in order to estimate the effect of the state of emergency (again captured as a binary variable for Post-April 1) on the probability of contraceptive receipt, controlling for a continuous time variable. For this analysis, the sample is not truncated and includes all women reported to receive facility referrals through May 20, the final day observed in the data. This model allows for censoring of data that arises given that some women have not yet visited a health facility at the close of the study. We present the estimated hazard ratios and standard errors, again clustered at the level of the week.

## Results

We begin by characterizing the sample of women observed in the data. 16% of women are below age 20; 32% are between age 20 and 24; 28% are between age 25 and 29; 21% are between age 30 and 39; and only 4% are over age 40. 62% of women report that they are already using contraceptives as of their first interaction with the promoter, and 27% report they have some access to a mobile phone.

**Fig 1:**
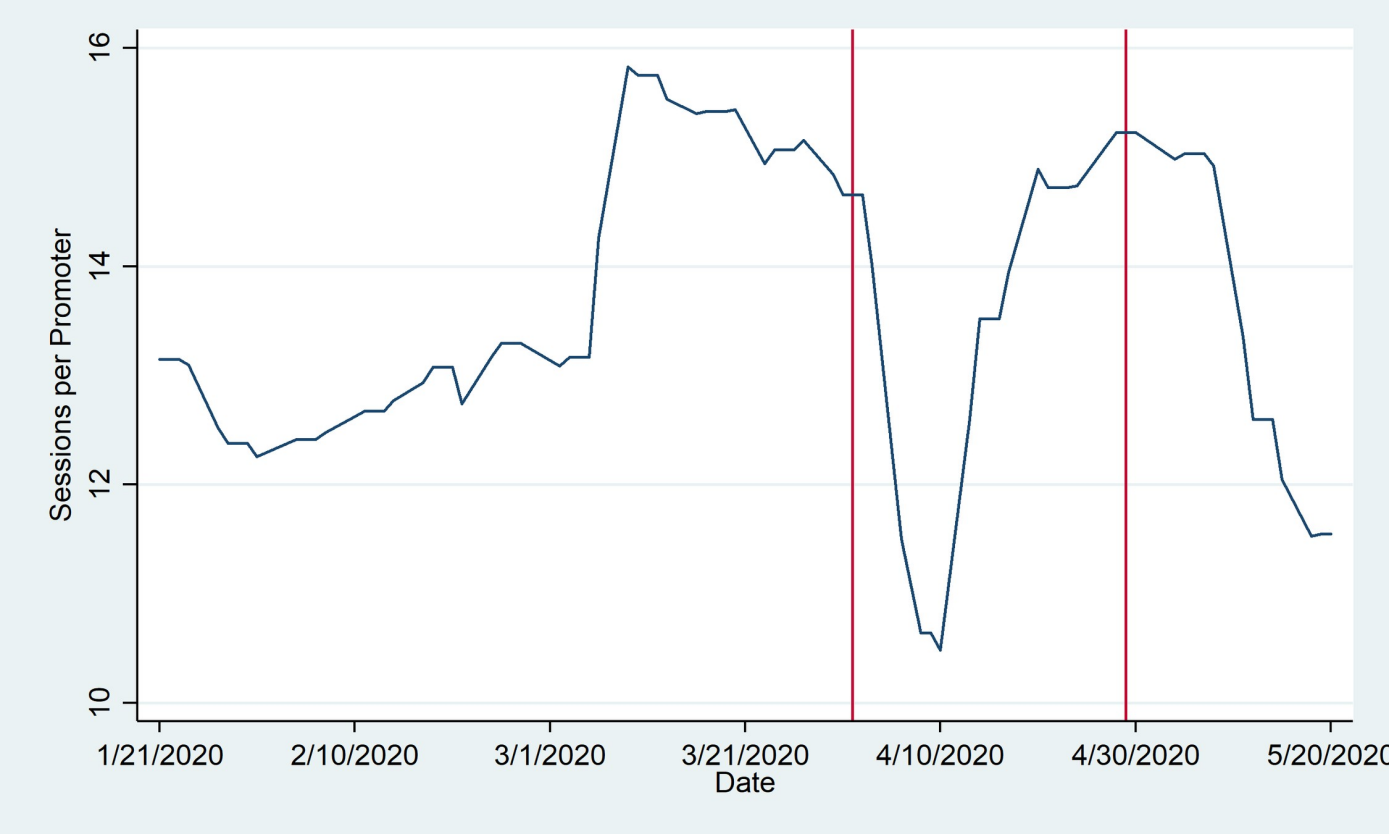
Daily sessions per promoter. Count of promoter sessions includes the sum of sessions with referrals, sessions without referrals, and reminder sessions by date conducted between January 21 and May 20, excluding weekends and public holidays (3-Feb, 7-Apr, and 1-May). These are normalized by the number of active promoters (i.e. promoters with at least one session) each day. Vertical lines denote the initial state of emergency declaration (1-Apr) and subsequent extension announcement (29-Apr). Trends are smoothed using a uniformly weighted moving average by week, taking into account the three days before and after in conjunction with the given date.

**Fig 2:**
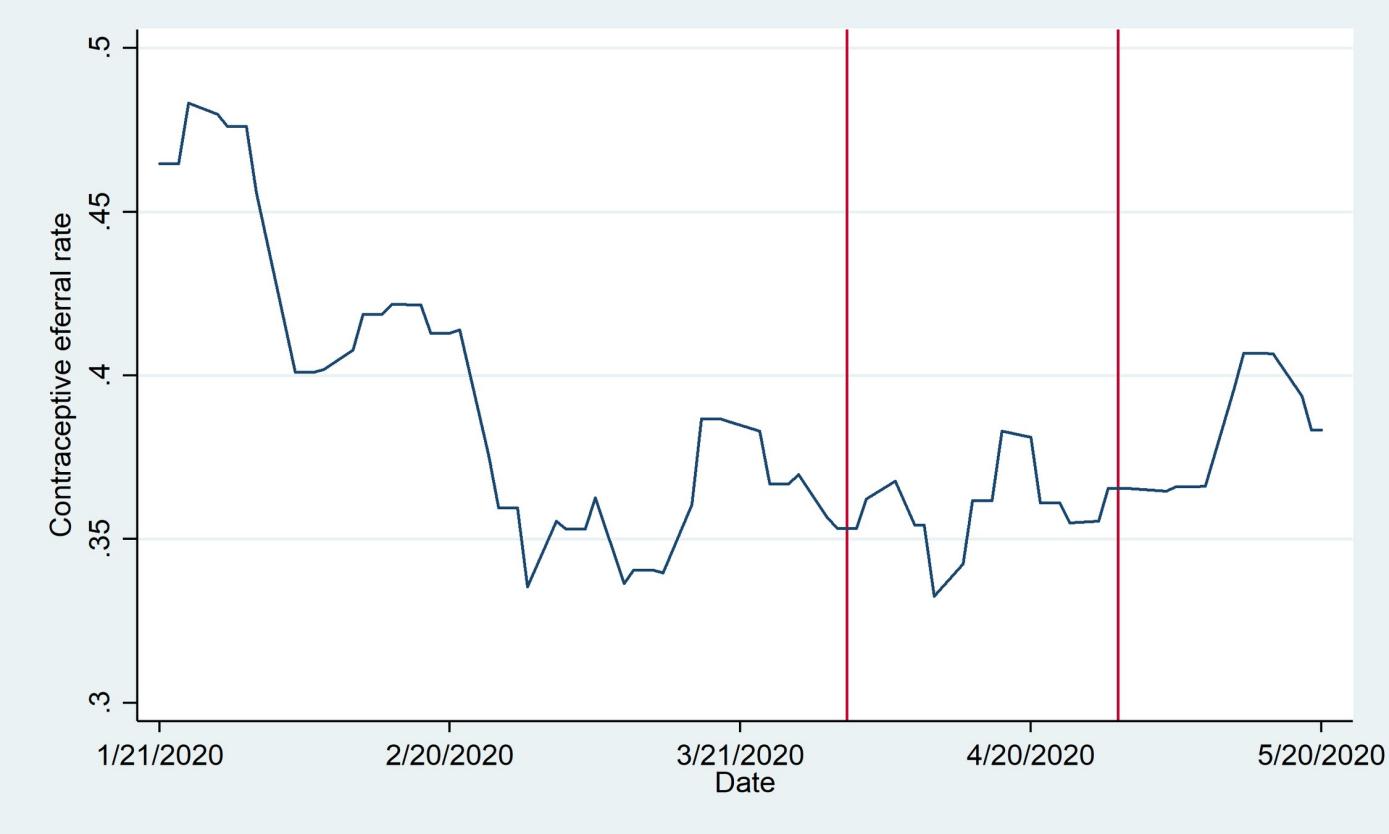
Referral rate by day of promoter visit. The referral rate is defined as the number of sessions in which referrals were issued as a proportion of sessions with and without referrals each day. The referral rate is reported for January 21 through May 20, excluding weekends and public holidays (3-Feb, 7-Apr, and 1-May). Vertical lines denote the initial state of emergency declaration (1-Apr) and subsequent extension announcement (29-Apr). Trends are smoothed using a uniformly weighted moving average by week, taking into account the three days before and after in conjunction with the given date.

Figs 1 and 2 illustrate the promoters’ behaviors. In Fig 1, it is evident that a substantial decline in the daily pace of promoter sessions was observed immediately followed the initial state of emergency, with the trough around April 8–9. However, this is followed by a rapid rebound through early May, when the daily pace of sessions began to decline again. More specifically, the daily average number of visits declined by 14% between April 1 and 15 relative to the month of March, but the decline moderates to about 10% following April 15; we observe an average of 14.8 sessions conducted per promoter per weekday in the month of March, 12.7 daily between April 1 and April 15, and 13.2 daily between April 15 and May 20. (Importantly, the number of active promoters was almost entirely consistent comparing the pre and post period: 116 promoters were active prior to April 1, and 122 promoters following April 1.)

In contrast to the pattern observed for promoter activity overall, Fig 2 demonstrates no clear pattern in the facility referral rate (averaging 37%) relative to the state of emergency. The referral rate generally was higher in the first weeks of the year, potentially reflecting a more intense period of activity following a hiatus in January. Although it diminished through the end of February, it was fairly constant through the period of emergency declaration and renewal.

**Fig 3:**
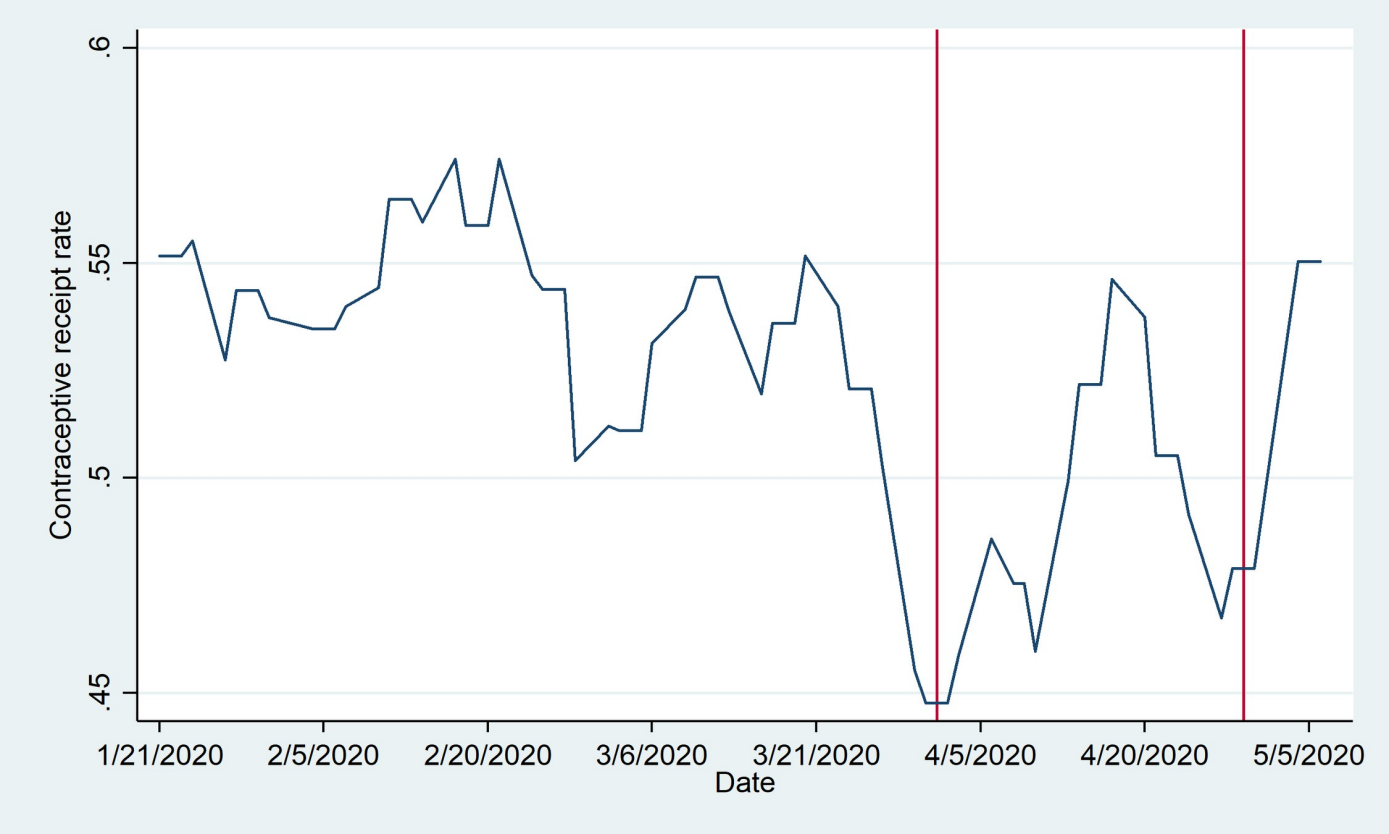
Contraceptive receipt rate. The contraceptive receipt rate is defined as the number of referred women who access a contraceptive method within 14 days of receiving the referral from a promoter as a proportion of all women who receive referrals from promoters that day. The uptake rate is reported according to the date of promoter visit between January 21 and May 20, excluding weekends and public holidays (3-Feb, 7-Apr, and 1-May). Vertical lines denote the initial state of emergency declaration (1-Apr) and subsequent extension announcement (29-Apr). Trends are smoothed using a uniformly weighted moving average by week, taking into account the three days before and after in conjunction with the given date.

While promoters demonstrated little change in behavior in response to the state of emergency, female participants in the program responded more robustly, as shown in Fig 3. Again, the date of reference is the date of the promoter visit; accordingly, a decline is observed in the days prior to April 1, reflecting the fact that women who received a referral in this period would have been visiting the facility around the declaration of the state of emergency and likely experienced a reduced propensity to utilize services. However, a rebound is observed by the middle of April, with a similar decline and rebound appearing around the date of the emergency extension on April 29. The low point of the contraceptive receipt rate (28%) is observed for referrals issued precisely on April 1, with a high point of around 60% reached by approximately April 20. This is followed by a more moderate decline to around 42% by the final week of April, and a subsequent rebound.

Table 1 presents the first set of regression results. In Columns (1) and (2), we observe that there is no significant association between a binary variable for contraceptive referral and a binary variable for post-April 1. In Columns (3) and (4), there is evidence of a significant association between a binary variable for contraceptive receipt and the binary post variable, particularly in the first two weeks of April relative to the previous month of March (OR 0.798, 95% CI [0.701–0.908], p = .001).

**Table 1:** Associations between post-April 1 and contraceptive referral / contraceptive receipt.

	(1)	(2)	(3)	(4)
	Contraceptive referral		Contraceptive receipt	
Post-April 1	0.921	0.927	0.889*	0.798***
	(0.813–1.042)	(0.805–1.067)	(0.782–1.012)	(0.701–0.908)
Observations	109,129	39,473	35,828	13,762
Dates	1/21-5/20	1/21-5/20	1/21-5/20	1/21-5/20

Notes: The table reports unadjusted odds ratios from logistic regressions, as well as the 95% confidence interval. The outcome variables are dichotomous variables for contraceptive referral and contraceptive receipt. The independent variable is a binary variable for post-April 1. Standard errors are clustered at the level of the week. Asterisks indicate significance at the ten, five and one percent level.s

**Fig 4:**
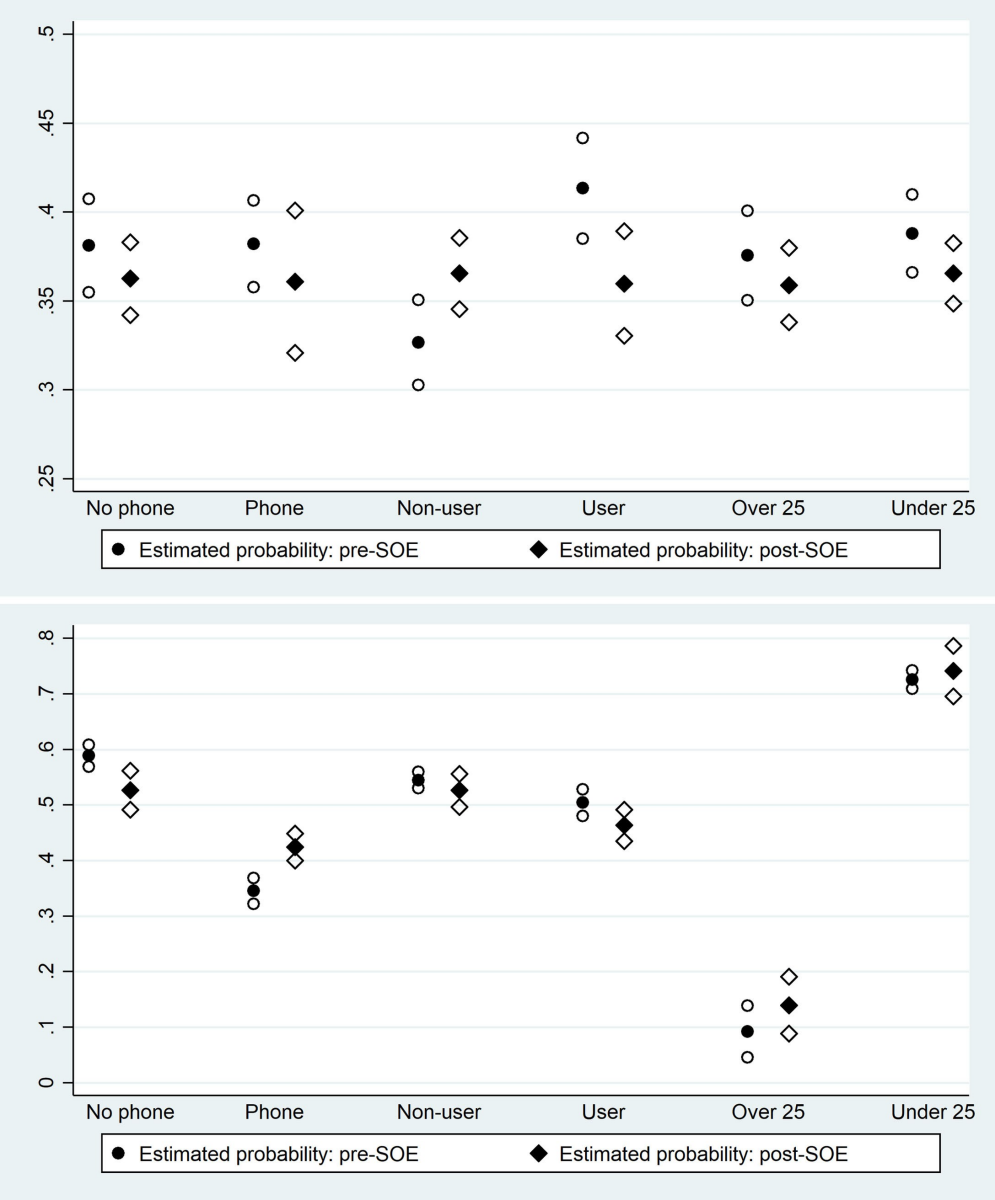
Predicted probabilities of contraceptive referral (4a) and contraceptive receipt (4b). These graphs show the predicted probabilities of contraceptive referral (Fig 4A) and contraceptive receipt (Fig 4B) corresponding to the logistic regressions presented in Table 2. Each graph presents the predicted probabilities for six subsamples of women: women who do and do not report access to a phone; women who are and are not current contraceptive users; and women under age 25 and over age 25. Predicted probabilities in the pre and post period are shown, in conjunction with 95% confidence intervals.

Table 2 presents the second set of regression results, analyzing interaction effects for access to a phone, age, and current use of contraception. In addition to the estimated odds ratios, the predicted probabilities of the outcome of interest (contraceptive referral / receipt) for women characterized by a value of zero or one for the individual characteristic of interest in the pre / post period are presented at the bottom of the table, where the characteristic itself is denoted X. (These predicted probabilities are estimated using the Stata command margins.) The predicted probabilities in conjunction with 95% confidence intervals are also presented in Fig 4.

**Table 2:** Associations between post-April 1 and contraceptive referral / contraceptive receipt: Interaction effects.

	(1)	(2)	(3)	(4)	(5)	(6)
	Contraceptive referral			Contraceptive receipt		
Post-COVID emergency	0.924	1.187**	0.931	0.777***	1.598	0.928
	(0.801–1.065)	(1.034–1.364)	(0.809–1.071)	(0.660–0.913)	(0.791–3.226)	(0.812–1.061)
Post X Reports access to phone	0.988			1.800***		
	(0.783–1.248)			(1.469–2.205)		
Reports access to phone	1.004			0.368***		
	(0.890–1.133)			(0.328–0.414)		
Post X Current contraceptive user		0.671***			0.677	
		(0.543–0.831)			(0.346–1.325)	
Current contraceptive user		1.453***			26.12***	
		(1.270–1.663)			(14.86–45.91)	
Post X Age under 25			0.976			0.914
			(0.902–1.057)			(0.815–1.026)
Age under 25			1.054*			0.850***
			(0.991–1.121)			(0.781–0.925)
Observations	109,129	109,129	109,129	35,828	35,828	35,828
Dates	1/21-5/20	1/21-5/20	1/21-5/20	1/21-5/20	1/21-5/20	1/21-5/20
**Predicted probabilities**						
Pre (X = 0)	0.381	0.327	0.376	0.589	0.545	0.092
Post (X = 0)	0.363	0.366	0.359	0.526	0.526	0.139
Pre (X = 1)	0.382	0.413	0.388	0.345	0.504	0.726
Post (X = 1)	0.361	0.360	0.366	0.424	0.463	0.741
X variable	Phone access	Current user	Age under 25	Phone access	Current user	Age under 25

Notes: The table reports unadjusted odds ratios from logistic regressions, as well as the 95% confidence interval. The outcome variables are dichotomous variables for contraceptive referral and contraceptive receipt. The independent variable is a binary variable for post-April 1, interacted with three individual-level characteristics. Standard errors are clustered at the level of the week. Predicted probabilities of contraceptive referral / receipt in the pre / post period for women in each subgroup are reported in the final five rows. Asterisks indicate significance at the ten, five and one percent level.

The regressions employing contraceptive referral as an outcome variable are reported in Columns (1) through (3). The only significant results are observed in Column (2), where both the estimated coefficient for Post-April 1 (OR 1.187, 95% CI [1.034, 1.354], p = .015) and the estimated interaction term with current user (OR 0.671, 95% CI [0.543, 0.831], p < .001) are statistically significant. Examining the predicted probabilities, the probability of referral for a woman who is not a current user of contraception in fact increases in the post period, while the probability of referral for a woman who is a current user declines. The regressions employing contraceptive receipt are reported in Columns (4) through (6), and the only significant results are observed in Column (4), where both the estimated coefficient for Post-April 1 (OR 0.777, 95% CI [0.660, 0.913], p = .002) and the estimated interaction term with reported phone access (OR 1.800, 95% CI [1.469, 2.205], p < .001) are statistically significant. Again, examining the predicted probabilities, the probability of receipt for a woman who does not report phone access declines in the post period, while the probability of receipt for a woman who reports phone access increases.

To provide a more nuanced depiction of shifts in trends in the pre and post emergency period, Table 3 reports the interrupted time series analysis, including the estimated coefficients and standard errors. Column (1) presents the results for contraceptive referrals. The baseline time trend is not statistically significant (β = -.0003, p = .199). The onset of the state of emergency is followed by a shift downward in the estimated intercept post-April 1 (β = -.127, p < .001), in conjunction with a significant and positive increase in the trend (β = .0020, p < .001). Column (2) presents the results for contraceptive receipt. The baseline trend is significant and positive, suggesting receipt rates were increasing (β = .0008, p = .020), while the onset of the state of emergency is followed by an even larger shift downward in the estimated intercept that is significant at the ten percent level (β = -.350, p = .067), in conjunction with a significant and positive increase in the trend (β = .0041, p = .049).

**Table 3:** Interrupted time series analysis.

	(3)	(4)
	Contraceptive referral	Contraceptive receipt
Post-April 1	-0.127***	-0.350*
	(0.0223)	(0.179)
Baseline trend	-0.000268	0.000757**
	(0.000201)	(0.000297)
Post X Baseline trend	0.00200***	0.00405**
	-0.127***	-0.350*
Observations	109,129	35,828
Dates	1/21-5/20	1/21-5/20

Notes: The table reports coefficients from an interrupted time series analysis in which the outcome variables are regressed on a baseline time trend, a binary variable for post-April 1, and an interaction between the two. Standard errors are clustered at the level of the week. Asterisks indicate significance at the ten, five and one percent level.

Finally, we report a Cox proportional hazards model to estimate the effect of the Post-April 1 state of emergency on contraceptive receipt. The estimated hazard ratio associated with the binary variable for Post-April 1 is .941 (standard error .042, p = .049), suggesting a reduction in the probability of contraceptive receipt in the Post-April 1 period. The estimated hazard ratio associated with the days elapsed variable is 1.002 (standard error .001, p = .016).

## Discussion

This analysis suggests that in general, the effect of the COVID-19 related state of emergency on utilization of contraceptive services in the context of a health promoter program providing door-to-door referrals to public health facilities in urban Mozambique has been modest in the first two months of the emergency. This finding is similar to previous evidence of a minor shift in contraceptive use in response to the Zika outbreak in Brazil [24], with the caveat that the current pandemic is very different from the Zika outbreak. This evidence is also in contrast to recent reports that contraceptive use may be declining rapidly following COVID-19 related disruptions in other contexts such as Indonesia [25], and preliminary evidence of substantial disruption in the delivery of other forms of preventive care (e.g., childhood vaccines) in developing countries during the COVID-19 crisis [26, 27].

In interpreting the shifts in promoter behavior, the brief reduction in the number of sessions per promoter observed in Fig 1 may reflect an increase in time invested per session, concerns about disease transmission in face-to-face interactions, or other related shocks. Immediately following the state of emergency declaration, promoters were instructed to provide information about the pandemic in conjunction with their standard script about family planning, possibly increasing the duration of each visit. At the same time, they may have reduced the number of sessions conducted to reduce their own risk of exposure.

Despite this short-term shift, however, our analysis suggests that promoters continued to refer women to health clinics to access contraceptive methods and more formal counseling from nurses; in an interrupted time series analysis, there is evidence of a downward shift in the estimated intercept for the referral rate following April 1, but a positive shift in the trend. This is suggestive evidence that promoters’ family planning messaging was not eclipsed by the pandemic messaging. Rather, these patterns may support the potential for future synergies via the utilization of existing community health workers to disseminate critical public health information in conjunction with previously established work activities [28].

In addition, we observe that women who visit facilities almost uniformly take up contraception, both before and during the state of emergency. In theory, a state of emergency could affect the availability of contraceptive methods themselves at clinics as well as staff to provide methods. However, there is no evidence of such an effect in this context.

Our analysis of women’s responses also suggests that there is a short-term decrease in the contraceptive receipt rate. Given that the majority of women receiving contraceptives through the IFPP program are utilizing short-term methods (76% of women utilize injections or oral contraceptives), a decline in the receipt rate could have meaningful implications.

Importantly, the observed disruptions to both service provision and contraceptive receipt appear to be largely short-term, and the rebound is rapid. This may reflect two important points. First, the state of emergency was not as stringent in Mozambique as in other countries, and there were no limitations on local freedom of movement or clinic closures. Second, the short-term growth of COVID-19 cases in Mozambique was relatively slow. As of the final day observed in this analysis (May 20, 2020), only 146 cases and zero deaths were reported nationwide [9]. Both promoters and women may have responded to the initial state of emergency by sharply shifting their behavior in anticipation of a substantial disruption to daily life and increasing health risks from COVID-19 transmission. When these expected shocks did not materialize, they may have returned quickly to their previous patterns. (Promoters do show a subsequent second dip in the intensity of daily sessions that is somewhat hard to interpret.) This observed rebound may be very different in other contexts more acutely affected by COVID-19 where case counts in fact did rise rapidly.

At the same time, there is some evidence that the decline in contraceptive referrals and receipts is not uniform for all women in the post-emergency period. In contrast to our ex ante hypotheses, the predicted probability of contraceptive referral in fact increases for women who are not current users of contraception post-emergency, and the predicted probability of contraceptive receipt increases for women who report access to phones. These seemingly counterintuitive patterns may reflect behavioral responses to the state of emergency. On the side of promoters, promoters may perceive that facility visits are more risky or inconvenient during the emergency period, and thus may shift their referrals toward women they perceive to be at higher risk of unintended pregnancy–women who are not using contraception at all–increasing the probability of referral for this subgroup.

On the side of women receiving services, we can hypothesize that women who have access to phones have more information about the COVID-19 pandemic and the anticipated health or economic risks, and increase their demand for contraception. Women who do not have access to phones do not show any comparable effect. Similarly, women who were previously non-users of contraceptives may also have shifted their fertility intentions during the state of emergency and thus received more referrals, though we do not have any direct measurement of fertility intentions. Our findings of differential patterns for women who do not report access to mobile phones echo the caution provided in Greenleaf et al. 2018 that reliance on cell-phone based interventions may be risky given their potentially limited reach in some contexts [29]. In regions where use of mobile phones by women and girls is higher, by contrast, these interventions may be more promising.

This analysis has a number of limitations, and among the most important is the limited data available. While using administrative data has notable advantages, including rapid access to the data and access to the full sample of program participants, one key disadvantage is that only very minimal data is collected. In particular, no data is available on women’s health history, sociodemographic characteristics, and preferences around fertility. Accordingly, we are unable to adjust the empirical analysis for these important covariates.

Additional limitations include that the analysis focuses on only prescribed contraception provided in public health facilities in a specific setting (two urban and peri-urban regions of Mozambique), and focuses on one particular model of service delivery via community health promoters and public clinics. Promoters in the IFPP program are also specifically incentivized to encourage women to visit clinics for contraceptive services, and thus may have continued to provide such encouragement and support during the state of emergency; this pattern may not be observed in other contexts.

Moreover, the state of emergency in Mozambique was not as stringent as those imposed in other settings, and the effects of stricter states of emergency may have been different. Accordingly, these findings are not necessarily generalizable for the provision of contraceptive or reproductive health services in other contexts, or for the provision of other forms of preventive health services.

## Conclusion

Overall, our findings suggest that the adverse effects of a state of emergency linked to COVID-19 on the delivery of contraceptive services via a community-based health promoter program were modest and short-term. In this context of urban Mozambique, a door-to-door method of service delivery for reproductive health services does not seem particularly vulnerable to the introduction of a state of emergency. There is, however, evidence of heterogeneous effects across women, including differential effects for women who have access to cell phones.

This evidence is informative about a particular service provision model for a particular population, but has clear limits in its relevance vis-a-vis other forms of reproductive health and preventive health services provided in other contexts. Given the uncertain duration of pandemic disruptions, it remains important to identify resilient models of service delivery that can also reach relatively more vulnerable populations.
