# Correction: Short-term effects of the COVID-19 state of emergency on contraceptive access and utilization in Mozambique

**DOI:** 10.1371/journal.pone.0317173

**Published:** 2025-01-03

**Authors:** Jessica Leight, Catherine Hensly, Marcos Chissano, Liza Ali

In Table 1, the reference period for the results indicated in the last row are incorrect. Please see the correct Table 1 here.

**Table 1:** Associations between post-April 1 and contraceptive referral / contraceptive receipt.

	(1)	(2)	(3)	(4)
	Contraceptive referral		Contraceptive receipt	
Post-April 1	0.921	0.927	0.889*	0.798***
	(0.813–1.042)	(0.805–1.067)	(0.782–1.012)	(0.701–0.908)
				
Observations	109,129	39,473	35,828	13,762
Dates	1/21-5/20	3/1-4/15	1/21-5/20	3/1-4/15

Notes: The table reports unadjusted odds ratios from logistic regressions, as well as the 95% confidence interval. The outcome variables are dichotomous variables for contraceptive referral and contraceptive receipt. The independent variable is a binary variable for post-April 1. Standard errors are clustered at the level of the week. Asterisks indicate significance at the ten, five and one percent level.
